# Anesthesia Medication’s Impacts on Inflammatory and Neuroendocrine Immune Response in Patients Undergoing Digestive Endoscopy

**DOI:** 10.3390/clinpract14030093

**Published:** 2024-06-18

**Authors:** Denisa-Ancuța Popa-Ion, Lidia Boldeanu, Dan-Ionuț Gheonea, Madalina Maria Denicu, Mihail Virgil Boldeanu, Luminița Cristina Chiuțu

**Affiliations:** 1 Department of Anesthesiology and Intensive Care, Faculty of Medicine, University of Medicine and Pharmacy of Craiova, 200349 Craiova, Romania; denisa.popa20@yahoo.com (D.-A.P.-I.); magda_ptre@yahoo.es (M.M.D.); luminita.chiutu@umfcv.ro (L.C.C.); 2 Department of Microbiology, Faculty of Medicine, University of Medicine and Pharmacy of Craiova, 200349 Craiova, Romania; 3 Department of Gastroenterology, Faculty of Medicine, University of Medicine and Pharmacy of Craiova, 200349 Craiova, Romania; 4 Department of Immunology, Faculty of Medicine, University of Medicine and Pharmacy of Craiova, 200349 Craiova, Romania; mihail.boldeanu@umfcv.ro

**Keywords:** catecholamines, interleukins, serum concentration, endoscopic procedure, anesthetic drugs

## Abstract

The aim of this study was to explore the impact of anesthetic drugs currently used to perform lower digestive endoscopy on serum concentrations of inflammation markers and catecholamines. We selected 120 patients and divided them into three lots of 40 patients each: L1, in which no anesthetics were used; L2, in which propofol was used; and L3, in which propofol combined with fentanyl was used. All patients had serum concentrations of adrenaline/epinephrine (EPI), noradrenaline/norepinephrine (NE), tumor necrosis factor alpha (TNF-α), interleukin-4 (IL-4), IL-6, IL-8, and IL-10, taken at three time points: at the beginning of the endoscopic procedure (T_0_), 15 min after (T_1_), and 2 h after the end of the endoscopic procedure (T_2_). The results of the research showed changes in the levels of catecholamines and interleukins (ILs) at T_0_, with an increased response in L1 above the mean recorded in L2 and L3 (*p* < 0.001). At T_1_, increased values were recorded in all lots; values were significantly higher in L1. At T_2_, the values recorded in L3 were significantly lower than the values in L2 (student T, *p* < 0.001) and L1, in which the level of these markers continued to increase, reaching double values compared to T_0_ (student T, *p* < 0.001). In L2 at T_1_, the dose of propofol correlated much better with NE, EPI, and well-known cytokines. Our results show that propofol combined with fentanyl can significantly inhibit the activation of systemic immune and neuroendocrine response during painless lower digestive endoscopy.

## 1. Introduction

Lower digestive endoscopy is a minimally invasive, short-to-medium duration procedure that causes pain, anxiety, vasovagal reactions, and implicit changes in the body’s inflammatory and neuroendocrine immune response [1,2,3]. 

Pain during the colonoscopy occurs due the air distension maneuvers of the colon and traction of the mesocolon, which is why patients scheduled for lower digestive endoscopy request it to be performed under sedation or analgosedation [4,5]. 

Endoscopy is the most common medical procedure performed under sedation in many countries. Lower digestive endoscopy, although a minimally invasive procedure, represents a determining stress factor in the appearance of the inflammatory and neuroendocrine immune response, resulting in an increase in the serum levels of pro- and anti-inflammatory cytokines and catecholamines. The anesthesia techniques used must be safe and must aid in decreasing the body’s immune response.

Prior research shows that anesthetic drugs, such as propofol and fentanyl, have an effect on the central nervous system and also on the endocrine and immune inflammatory system, attenuating the release of various types of cytokines and catecholamines into the blood flow and thus conferring protection on the immune reaction triggered by the endoscopic procedure [6,7]. 

Anesthetic drugs may be associated with postprocedural immunosuppression, either directly by activating immune mediating cells or indirectly by modulating the stress response. Studies have investigated which anesthetics may alter the functions of immunocompetent cells and change the expression of inflammatory mediator genes [8,9,10,11]. 

The release of chemical mediators in the bloodstream is triggered by the digestive endoscopic procedure itself, and the most frequently encountered markers of inflammatory and neuroendocrine immune response are represented by the interleukins 4, 6, 8, and 10, along with TNF-α and catecholamines [12,13,14,15]. 

Catecholamines are stress hormones that act on all tissues, exerting important effects on cardiac, metabolic, endocrine, and neurological functions, affecting the intestinal barrier and, consequently, the immune response [16,17,18,19].

Recent studies have shown that the simultaneous release of pro- and anti-inflammatory cytokines is necessary for any immune response to maintain a balance [15,20]. Under normal conditions, pro- and anti-inflammatory cytokines serve as key immunomodulatory elements that limit injuries and extreme inflammatory reactions. Under pathological conditions, their imbalance may cause an excessively high or excessively low systemic inflammatory response [21,22,23,24]. Drugs such as propofol and fentanyl have been shown to potentially reduce the release of inflammatory cytokines and vasoactive hormones [13,25].

The purpose of this prospective study was to demonstrate that the use of analgosedation (propofol plus fentanyl) during a colonoscopy, in addition to increasing intraprocedural comfort, acts on cytokine and catecholamine levels by decreasing the inflammatory and neuroendocrine immune response.

## 2. Materials and Methods

Collection of the patients’ data

**Figure 1:**
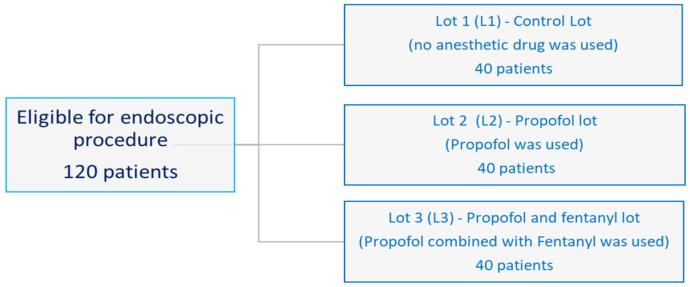
A flow chart for including patients.

This is a prospective, double-blind, randomized study. One hundred and twenty patients who underwent lower digestive endoscopy at the Hepatology and Gastroenterology Research Centre, University of Medicine and Pharmacy of Craiova (UMPh Craiova), from March 2023 to October 2023, were recruited and divided using a random number table into three lots of 40 patients. The 40 patients in L1 (control lot) received no anesthetic or sedative drugs; the 40 patients in L2 (propofol lot) were anesthetized with propofol; and the 40 patients in L3 (propofol and fentanyl lot) were anesthetized with propofol and fentanyl (Figure 1).

Inclusion and exclusion criteria

Inclusion criteria: patients with American Society of Anesthesiologists (ASA) Physical Status I and II [26], patients scheduled for lower digestive endoscopy, patients aged between 18 and 80 years, patients diagnosed with inflammatory bowel disease, patients diagnosed with colorectal cancer.

Exclusion criteria: patients with cardiac pathology combined with decompensated respiratory or other pathology, patients with severe allergies to the anesthetic drugs used, pregnant female patients, patients who required long-term use of various sedative drugs due to underlying disease before inclusion in this study, patients scheduled for upper gastrointestinal endoscopies (as the duration of these procedures may be too short to change the value of the studied parameters).

All patients consented to this study and signed an agreement. This study was approved by the Ethics Committee of UMPh Craiova, no. 37/1st March 2022, in accordance with the Declaration of Helsinki.

Anesthesia methods

In L2, we used 2 mg/kg body weight Fresenius Kabi propofol 10 mg/mL in 20 mL vials (Fresenius Kabi Deutschland GMBH) for anesthetic induction, followed by injection of an additional dose of 10–20 mg propofol after a 5 min interval. We did not use infusion pumps to administrate propofol because the examination time was short (15–20 min); to maintain vital functions within the parameters, two or three bolus administrations were necessary, depending on the purpose of the procedure.

For L3, we used Kalceks fentanyl 50 micrograms/mL in 10 mL vials (Akcju Sabiedriba Kalceks, Latvia), 0.01 micrograms/kg body weight, administered 5 min before the start of the procedure, along with 1.5–2 mg/kg body weight Fresenius Kabi propofol 10 mg/mL in 20 mL vials (Fresenius Kabi Deutschland GMBH) for anesthetic induction.

In L3 patients for which the duration of lower digestive endoscopy was increased due to therapeutic procedures (polypectomies, biopsies), propofol redosing was necessary.

All patients received pure oxygen 6 L/min flow through the nasal cannula from the beginning of lower digestive endoscopy. Patients included in this study had stable vital functions throughout their procedures, without the need for intubation or transfer to an intensive care unit. The actual endoscopic procedures began when the ciliary reflex disappeared.

The Richmond Agitation Sedation Scale (RASS) [27,28], with four levels of anxiety or agitation, and the Modified Observer’s Assessment of Alertness and Sedation (MOAA/S) scale [29,30] were used to assess the depth of sedation. If the MOAA/S scale score was >3, a dose of 20 mg propofol was administered to maintain anesthetic sleep. 

Each patient’s endoscopic procedure was performed for screening, diagnostic, and therapeutic purposes, by the same gastroenterologist, with at least 10 years of experience in the field, using standard colonoscopy equipment, an Olympus EVIS X1CV-1500 CF-EZ1500DL 3.7. All colonoscopies were performed completely, with visualization of the ileocecal valve.

Monitoring of vital cardiovascular parameters was performed indirectly using the GE B125 VSP 2.0 portable monitor, which includes an automatic sphygmomanometer, and consisted of blood pressure measurement every 5 min until the end of the intervention and a finger pulse oximeter to monitor peripheral tissue oxygen saturation and ventricular allure.

Venous access was performed by applying a 16-gauge diameter flexure placed at the forearm for injection of anesthetic solutions.

### 2.1. Blood Collection

Peripheral venous blood was collected at 3 different times: at the beginning of the procedure and administration of anesthetic drugs (T_0_), 15 min after the beginning of the endoscopic procedure (T_1_), and 2 h after the end of the colonoscopy (T_2_). Blood samples were collected on EDTA 6 mL from each patient and centrifuged at 5000 rpm for 5 min 24 h after collection. Plasma was stored at −80 degrees Celsius until testing. 

### 2.2. Immunological Determinations of Serum Markers

Samples were coded and analyzed in the Immunology Laboratory of the UMPh of Craiova, using the enzyme-linked immunosorbent assay (ELISA) technique to detect the concentration levels of catecholamines and well-known cytokines.

The ELISA kits were purchased from Elabscience (Houston, TX, USA): TNF-α (cat. no.: E-EL-H0109; sensitivity: 4.69 pg/mL; detection range: 7.81–500 pg/mL); IL-6 (cat. no.: E-EL-H6156; sensitivity: 0.94 pg/mL; detection range: 1.56–100 pg/mL); IL-8 (cat. no.: E-EL-H6008; sensitivity: 4.69 pg/mL; detection range: 7.81–500 pg/mL); IL-4 (cat. no.: E-EL-H0101; sensitivity: 18.75 pg/mL; detection range: 31.25–2000 pg/mL); IL-10 (cat. no.: E-EL-H6154; sensitivity: 0.94 pg/mL; detection range: 1.56–100 pg/mL); NE (cat. no.: E-EL-H0047; sensitivity: 0.19 ng/mL; detection range: 0.31–20 ng/mL); EPI (cat. no.: EL-H0045; sensitivity: 18.75 pg/mL; detection range: 31.25–2000 pg/mL). 

### 2.3. Statistic Methods

Data were collected and analyzed using Microsoft Excel 2019 (Microsoft, Raymond, Washington, DC, USA). The analytical procedures encompassed descriptive statistics, which included frequency analysis, computation of statistical indicators, and visualizations. Given the exclusive focus on numerical data, inferential statistics were conducted partly within Excel and partly using MATLAB (Mathworks, Natick, MA, USA).

The assessment of data normality was performed utilizing the Shapiro–Wilk test. Since all resultant data exhibited a Gaussian distribution, analysis of variance (ANOVA) and Student’s *t*-test were employed for multiple and two-sample data, respectively. Significance was determined at a *p*-value threshold of less than 0.05, and values below 0.001 were denoted as *p* ≤ 0.001.

No post-hoc analysis was conducted because our focus was on making paired comparisons between two groups at a time. Therefore, the primary statistical test used for this purpose was the Student’s *t*-test. The ANOVA was employed primarily to confirm and support the findings highlighted by the individual *t*-tests. 

Using Pearson’s coefficients (−1 < r < 1), significant correlations between catecholamine levels (NE and EPI) and inflammatory state biomarkers (TNF-α, IL-6, IL-8, IL-4, and IL-10) were assessed. The results were shown visually using a correlation heatmap matrix, where high positive correlations were represented by bright green and strong negative correlations by blazing red.

## 3. Results

The demographics data are summarized in Table 1. There were no significant differences between the lots in patient clinical characteristics.

There were no significant differences between the groups in the distribution of therapeutic interventions; there were two biopsies and one polypectomy performed in L1, four polypectomies in L2, and four polypectomies and one biopsy in L3.

The mean ± SD dose of propofol used in L2 was 272.00 ± 59.25 mg. In L3, the dose of propofol was significantly lower, with a mean of 164.50 ± 25.43 mg.

The RASS score (mean ± SD) for the two lots where we used anesthetic drugs was −4.90 ± 0.30 in L3 and −3.10 ± 1.20 in L2 (statistically significant, *p* < 0.001). 

The duration (mean ± SD) of the endoscopic procedure in minutes in each lot studied was as follows: 23.10 ± 3.69 in L1, 17.75 ± 2.61 in L2, and in L3, the shortest examination time, 15.10 ± 1.94. The difference in the times of the three lots was statistically significant (*p* < 0.001). 

In L2, where we used propofol for sedation, patients had a shorter wake-up time (mean ± SD) of 3.30 ± 0.90 min, with a statistically significant difference (*p* < 0.001), compared to L3 where the wake-up time (mean ± SD) was 5.20 ± 1.00 min, due to the cumulative effect of the two anesthetics drugs.

### 3.1. Concentrations of Serum Catecholamines and Cytokines

Table 2 shows the changes in the concentration of stress indicators at different time points. It can be seen that catecholamine level remains unchanged at T_0_ in the two lots where anesthetic drugs were used. L1 shows a higher level compared to the two lots. This level continues to increase at T_1_ in L1 and L2, and at T_2_, it is significantly lower in L3 than in the other two lots.

The serum concentrations of pro-inflammatory cytokines (IL-6, IL-8, and TNF-α) in the three lots are detailed in Table 3. At T_0_, the levels of IL and TNF-α concentration were significantly different in L2 and L3, with a higher value in L1. At T_1_, they were increased in all lots, especially in the L2 and L1. At T_2_, they were significantly lower L3 than in L2 and L1.

Comparison of anti-inflammatory cytokines IL-4 and IL-10 in the lots studied at different time intervals was as follows: at the beginning of the endoscopic intervention (T_0_), statistical changes in the plasma concentration of the two cytokines were observed in the three lots, with significant differences between L1 and L3. At 15 min after the beginning of the endoscopic intervention (T_1_), an explosive increase in serum concentration was observed in the L1, as well as a moderate increase in L2 and L3. However, there was a significantly greater decrease 2 h after the end of the endoscopic digestive procedure (T_2_) in L3 compared to L2 and L1. Results are shown in the Table 4.

### 3.2. Catecholamine and Cytokine Levels Correlated Much Better with the Dose of Propofol in L2

In L3 (propofol combined with fentanyl) at T_1_ (15 min after the beginning of the endoscopic procedure), Pearson’s test indicated a statistically significant correlations between dose of propofol and EPI (weak correlation, r = 0.294, *p* = 0.017), and IL-8 levels (weak correlation, r = 0.215, *p* = 0.048). 

The TNF-α level correlated moderately and significantly with the NE levels (r = 0.468, *p* = 0.002), and weakly but significantly with the EPI levels (r = 0.279, *p* = 0.037). The EPI levels also correlated positively with IL-10 levels (weak correlation, r = 0.246, *p* = 0.028), and correlated negatively with IL-6 levels (moderate correlation, r = −0.317, *p* = 0.043).

There was an important moderate correlation between TNF-α and IL-10 levels (r = 0.486, *p* = 0.001).

**Figure 2:**
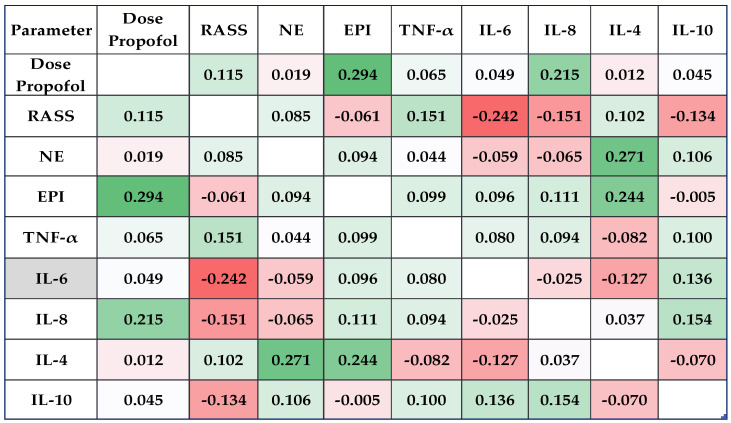
The correlation heatmap matrix between the measured catecholamines and cytokines (colors ranging from brilliant red for strong negative correlations to bright green for strong positive correlations) in L3 (lot that received propofol combined with fentanyl) at T_1_ (15 min after the beginning of the endoscopic procedure) time. RASS: Richmond Agitation Sedation Scale; TNF-α: tumor necrosis factor alpha; IL: interleukin; NE: norepinephrine; EPI: epinephrine.

The matrix correlation between the NE, EPI, TNF-α, IL-6, IL-10, and RASS values and dose of propofol is presented in Figure 2.

**Figure 3:**
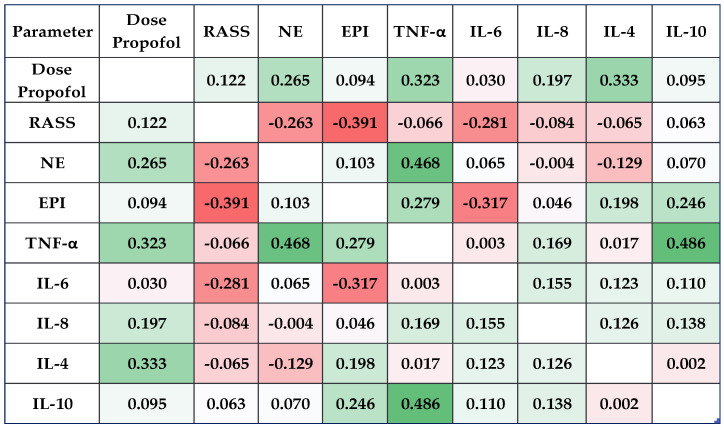
The correlation heatmap matrix between the measured catecholamines and cytokines (colors ranging from brilliant red for strong negative correlations to bright green for strong positive correlations) in L2 (lot in which propofol was used) at T_1_ (15 min after the beginning of the endoscopic procedure). RASS: Richmond Agitation Sedation Scale; TNF-α: tumor necrosis factor alpha; IL: interleukin; NE: norepinephrine; EPI: epinephrine.

In the L2 lot (propofol), Pearson’s test showed that the dose of propofol correlated positively with the NE (weak correlation, r = 0.265, *p* = 0.031), TNF-α (weak correlation, r = 0.323, *p* = 0.023), and IL-4 levels (weak correlation, r = 0.333, *p* = 0.040). Other significant positive correlations were found between IL-4 and NE levels (weak correlation, r = 0.2471, *p* = 0.002), and EPI levels (weak correlation, r = 0.244, *p* = 0.004) (Figure 3).

## 4. Discussion

Unhealthy eating habits, environmental factors, and the increasing incidence of inflammatory or tumoral gastrointestinal diseases have recently led to an increase in the number of endoscopic procedures performed for diagnostic and therapeutic purposes [27,28]. 

When exposed to stress, the body responds by increasing sympathetic nervous system activity, resulting in the release of neuroendocrine hormones called catecholamines (EPI, NE) and pro- and anti-inflammatory cytokines (IL-4, IL-6, IL-8, IL-10, TNF-α) into the circulation [6,24,29,30,31].

Out of the numerous cytokines, the current research only studied IL-4, IL-6, IL-8, IL-10, TNF-α, and catecholamines (EPI, NE). At a predetermined time, we followed up on their evolution independent of any drug influence (L1) and their evolution under the influence of drugs commonly used for endoscopic procedures (L2 and L3).

Fentanyl has a dose-dependent immunosuppressive effect, crossing the blood–brain barrier and exerting an immunomodulatory effect under stress conditions affecting cytokine release, lymphocyte proliferation, macrophage proliferation, and monocyte and neutrophil functions [29]. In low doses, it can be used in short-term exploratory or therapeutic procedures, with increased analgesic effect [1,32].

Propofol is a hypnotic agent considered to have immunomodulatory properties, acting on pro- and anti-inflammatory cytokines and producing favorable effects on the body by inhibiting cell apoptosis and inflammation and maintaining ionic homeostasis [33,34,35,36,37,38,39,40,41]. It inhibits cyclooxygenase 2, reduces the production of prostaglandin E2, preserves the function of natural killer cells, decreases the production of pro- and anti-inflammatory cytokines, and enhances the activation and peripheral differentiation of T helper cells [16,42].

Numerous studies have shown that a single dose of propofol used to induce anesthetic sleep (propofol 1.5–2 mg/kg body weight) suppresses the level of circulating EPI, NE, IL-4, IL-6, IL-8, IL-10, and TNF-α, but does not completely block their release [29,37,43,44,45,46,47,48,49,50].

In our study, we followed the impact of various sedative/hypnotic agents on the inflammatory and neuroendocrine immune response during a lower digestive endoscopy.

In L1 patients, who did not receive any analgosedation drugs, the results showed an increase in the plasma levels of IL-4, IL-6, IL-8, IL-10, and TNF-α at T_0_, T_1_, and T_2_. EPI had an ascending slope until T_2_, reaching a double value that was recorded at T_0_. NE had a pattern similar to that of EPI, with a steady increase in serum concentration at T_1_ and T_2_.

Therefore, in L1 patients who did not benefit from any anesthetic technique, we were able to highlight the real behavior and evolution of post-traumatic stress markers on the body. 

In L2, a peak was recorded at T_1_ in the basal value (T_0_) of all ILs and catecholamines. Their levels decreased at T_2_.

In L3, we started at time T_0_ with the determination of the serum concentration level of the inflammatory immune factors EPI, NE, IL-4, IL-6, IL-8, IL-10, and TNF-α, which initially increased until time T_1_, then gradually decreased. At T_2_, they reached lower values than those recorded at T_0._

This behavior occurs because analgosedation attenuates the body’s immune reaction to the stress induced by the endoscopic procedure more than propofol alone.

The severity of sedation level during procedures was much higher in patients in L3, as evidenced by the RASS score, which averaged −5, compared to patients in L2, who had an average RASS score of −3. We used this score because it appears to more accurately show the depth of sedation than using the Ramsay Sedation Scale (RSS) [51,52,53,54,55,56,57,58].

Several studies have shown an increased incidence of colorectal cancer in patients from the southern rural area of Romania, compared to those from urban areas. By comparing the number of patients from each area, we highlighted that the number of patients from rural areas requesting colonoscopies is still low (28.3%). Consequently, the incidence of colorectal cancer remains high in this population group, although in recent years, numerous screening programs for the early detection of lesions preceding colorectal cancer have been implemented.

One patient from L1 was diagnosed with chronic hepatitis C in his pathological personal history, but no viremia was detected. Another patient from L3 had inflammatory bowel disease (hemorrhagic rectocolitis). Thirteen patients included in this study were diagnosed with colorectal cancer; most (eight patients) were in L1, with three in L2 and two in L3. All these patients had IL and catecholamine levels similar to those who did not present neoplastic pathology in all the studied groups.

In our study, we observed that the average dose of propofol administered to the investigated patients correlated much better, positively, statistically significantly, and weakly with NE, TNF-α, and IL-4, in the L2 group, in contrast to the L3 group, where we found correlations positive and weak between the propofol dose, EPI, and IL-8. We did not find any studies in the literature that communicate the relationship between propofol doses and cytokine levels. According to our findings, we found that fentanyl in combination with propofol, the combination used in the L3 group, can modulate by reducing the systemic immune and neuroendocrine response during a painless lower gastrointestinal endoscopy. The data obtained in our study can constitute a starting point to investigate the direct effect of propofol and/or fentanyl doses on the level of catecholamines and cytokines.

We recognize that there are inherent limitations to this investigation, which was limited to our reference center. The time constraint imposed on this study—we chose to perform a prospective study over a period of 6 months as long as we had access to the respective internship. During the 6 months of this study, only 120 patients underwent a colonoscopy. Although we used 40 patients in each lot, we reached statistical significance, so with regard to the simulation of sample size determination, we consider it unnecessary at this point. We also did not calculate the sample size because we could not estimate the effect size, as we found no pilot or previous data mentioned in the literature. Resource constraints were exacerbated by the need to complete a doctoral thesis. 

Another limitation is that we did not have baseline values for all lots. We used L1 as a reference value in which we did not administer any anesthetic or sedative drugs.

Additionally, the patients included in this study received colonoscopies in outpatient clinics and were discharged on the same day, so we could not observe the dynamics of the level of ILs and catecholamines for a longer period of time (8 or 24 h post-colonoscopy).

The lack of equipment to monitor the depth of sedation is another limitation of our research. We resorted to assessing the degree of sedation depth using the RASS, because we did not have a BIS assessment monitor or Narcotrend monitor during this study [59,60,61].

Fentanyl combined with propofol has an inhibiting effect on systemic immune reaction during painless colonoscopy, and it can decrease the secretion of adrenal medullary hormones and inflammatory immune factors. For a better understanding of this complex process, the study remains open to future research. 

## 5. Conclusions

This study proved the presence of inflammatory and neuroendocrine immune response in patients undergoing outpatient lower digestive endoscopy and traced the dynamics of the markers studied during the investigation at well-defined time points in the absence and presence of analgosedation use.

The anesthetic induction of propofol combined with fentanyl can improve inflammatory and neuroendocrine immune response, shorten anesthesia time, and promote the recovery of post-procedural cognitive function. It should thus be extensively adopted.

As a final observation, analgosedation (propofol plus fentanyl) exhibits a high safety profile and causes lower serum concentration levels of IL-4, IL-6, IL-8, IL-10, TNF-α, EPI, and NE at 2 h after the end of an endoscopic procedure, which mitigates the immune response, reducing the risk of post-procedural complications and improving clinical outcomes. In L2 at T_1_, the dose of propofol correlated much better with NE, EPI, and well-known cytokines.

## Figures and Tables

**Table 1:** Clinical characteristics and patient demographics.

Parameters		L1 (*n* = 40)	L2 (*n* = 40)	L3 (*n* = 40)
Gender, male/female, (*n*)		21/19	21/19	18/22
Average age (standard deviation)		56.3 ± 16.7	59.2 ± 14.4	54.4 ± 15.1
Area of residence, urban/rural, (*n*)		14/26	11/29	9/31
ASA I, (*n*)		16	15	20
ASA II, (*n*)		24	25	20
Dose of propofol, (mean ± SD) (mg)		-	272.00 ± 59.25	164.50 ± 25.43
RASS score, (mean ± SD)		-	−3.10 ± 1.20	−4.90 ± 0.30
Duration of the endoscopic procedure, (min) (mean ± SD)		23.10 ± 3.69	17.75 ± 2.61	15.10 ± 1.94
Wake-up time, (min) (mean ± SD)		-	3.30 ± 0.90	5.20 ± 1.00
BMI, (*n*)	Normal weight	10	16	14
	Overweight	13	5	16
	Obesity	17	19	10
Comorbidities, (*n*)	Hypertension	11	9	12
	Diabetes	7	4	7
	Digestive cancer	8	3	2

ASA: American Society of Anesthesiologists; BMI: body mass index; L1: lot 1; L2: lot 2 with propofol; L3: lot 3 with propofol combined with fentanyl; RASS: Richmond Agitation Sedation Scale.

**Table 2:** Serum concentration of EPI and NE in the lots studied at different time intervals.

				*p*-Value			
EPI (pg/mL)				Student’s *t*-Test			One-Way ANOVA
Lot (*n* = 40)	T_0_	T_1_	T_2_	T_0_ vs. T_1_	T_0_ vs. T_2_	T_1_ vs. T_2_	
L1	606.0 ± 189.2	898.3 ± 274.0	1046.6 ± 261.5	<0.0001 ***	<0.0001 ***	0.015 *	<0.0001 ***
L2	534.3 ± 95.7	671.9 ± 119.2	465.1 ± 87.6	<0.0001 ***	0.001 **	<0.0001 ***	<0.0001 ***
L3	446.7 ± 147.9	522.2 ± 153.4	297.0 ± 115.0	0.028 *	<0.0001 ***	<0.0001 ***	<0.0001 ***
One-way ANOVA	<0.0001 ***	<0.0001 ***	<0.0001 ***				
L1 vs. L2 (*p*)	0.037 *	<0.0001 ***	<0.0001 ***				
L1 vs. L3 (*p*)	<0.0001 ***	<0.0001 ***	<0.0001 ***				
L2 vs. L3 (*p*)	0.002 **	<0.0001 ***	<0.0001 ***				
NE (ng/mL)							
L1	10.7 ± 1.9	12.4 ± 2.4	14.2 ± 2.4	0.001 **	<0.0001 ***	0.001 **	<0.0001 ***
L2	9.3 ± 2.1	10.8 ± 2.9	7.2 ± 1.8	0.010 *	<0.0001 ***	<0.0001 ***	0.004 **
L3	9.2 ± 1.4	8.7 ± 2.2	3.4 ± 1.2	0.040 *	<0.0001 ***	<0.0001 ***	<0.0001 ***
	T_0_	T_1_	T_2_				
One-way ANOVA	<0.0001 ***	<0.0001 ***	<0.0001 ***				
L1 vs. L2 (*p*)	0.004 **	0.009 **	<0.0001 ***				
L1 vs. L3 (*p*)	<0.0001 ***	<0.0001 ***	<0.0001 ***				
L2 vs. L3 (*p*)	<0.0001 ***	<0.0001 ***	<0.0001 ***				

EPI: adrenaline/epinephrine; NE: noradrenaline/norepinephrine; L1: lot 1; L2: lot 2 with propofol; L3: lot 3 with propofol combined with fentanyl; T_0_: at the beginning of the procedure and administration of anesthetic drugs; T_1_: 15 min after the beginning of the endoscopic procedure; T_2_: 2 h after the end of the colonoscopy; * *p*-value < 0.05; ** *p*-value < 0.01; *** *p*-value < 0.0001.

**Table 3:** Serum concentration of pro-inflammatory cytokines (IL-6, IL-8, and TNF-α) in the lots studied at different time intervals.

				*p*-Value			
IL-6 (pg/mL)				Student’s *t*-Test			One-Way ANOVA
Lot (*n* = 40)	T_0_	T_1_	T_2_	T_0_ vs. T_1_	T_0_ vs. T_2_	T_1_ vs. T_2_	
L1	8.3 ± 3.1	13.6 ± 2.9	16.4 ± 3.4	<0.0001 ***	<0.0001 ***	<0.0001 ***	<0.0001 ***
L2	6.4 ± 1.1	8.2 ± 1.5	5.1 ± 1.2	<0.0001 ***	<0.0001 ***	<0.0001 ***	<0.0001 ***
L3	5.4 ± 1.7	6.9 ± 2.6	3.1 ± 1.1	<0.0001 ***	0.003 **	<0.0001 ***	<0.0001 ***
One-way ANOVA	<0.0001 ***	<0.0001 ***	<0.0001 ***				
L1 vs. L2 (*p*)	<0.0001 ***	<0.0001 ***	<0.0001 ***				
L1 vs. L3 (*p*)	<0.0001 ***	<0.0001 ***	<0.0001 ***				
L2 vs. L3 (*p*)	0.002 **	0.007 **	<0.0001 ***				
IL-8 (pg/mL)							
L1	160.3 ± 40.5	243.3 ± 59.5	295.4 ± 56.8	<0.0001 ***	<0.0001 ***	<0.0001 ***	<0.0001 ***
L2	111.7 ± 23.0	138.7 ± 27.3	89.7 ± 14.4	<0.0001 ***	<0.0001 ***	<0.0001 ***	<0.0001 ***
L3	85.5 ± 36.2	119.0 ± 37.6	41.2 ± 16.1	<0.0001 ***	<0.0001 ***	<0.0001 ***	<0.0001 ***
	T_0_	T_1_	T_2_				
One-way ANOVA	<0.0001 ***	<0.0001 ***	<0.0001 ***				
L1 vs. L2 (*p*)	<0.0001 ***	<0.0001 ***	<0.0001 ***				
L1 vs. L3 (*p*)	<0.0001 ***	<0.0001 ***	<0.0001 ***				
L2 vs. L3 (*p*)	<0.0001 ***	0.009 **	<0.0001 ***				
TNF-α (pg/mL)							
L1	20.3 ± 6.7	24.2 ± 7.6	29.5 ± 8.3	<0.0001 ***	0.017 *	<0.0001 ***	0.004 **
L2	15.4 ± 1.9	16.5 ± 2.0	8.0 ± 1.4	<0.0001 ***	0.013 *	<0.0001 ***	<0.0001 ***
L3	13.9 ± 2.4	15.2 ± 2.7	3.2 ± 0.6	<0.0001 ***	0.021 *	<0.0001 ***	<0.0001 ***
	T_0_	T_1_	T_2_				
One-way ANOVA	<0.0001 ***	<0.0001 ***	<0.0001 ***				
L1 vs. L2 (*p*)	<0.0001 ***	<0.0001 ***	<0.0001 ***				
L1 vs. L3 (*p*)	<0.0001 ***	<0.0001 ***	<0.0001 ***				
L2 vs. L3 (*p*)	0.002 **	0.013 *	<0.0001 ***				

IL: interleukin; TNF-α: tumor necrosis factor alpha; L1: lot 1; L2: lot 2 with propofol; L3: lot 3 with propofol combined with fentanyl; T_0_: at the beginning of the procedure and administration of anesthetic drugs; T_1_: 15 min after the beginning of the endoscopic procedure; T_2_: 2 h after the end of the colonoscopy; * *p*-value < 0.05; ** *p*-value < 0.01; *** *p*-value < 0.0001.

**Table 4:** Comparison of anti-inflammatory cytokines IL-4 and IL-10 in the lots studied at different time intervals.

				*p*-Value			
IL-4 (pg/mL)				Student’s *t*-Test			One-Way ANOVA
Lot (*n* = 40)	T_0_	T_1_	T_2_	T_0_ vs. T_1_	T_0_ vs. T_2_	T_1_ vs. T_2_	
L1	102.8 ± 24.2	136.2 ± 26.9	154.8 ± 26.7	<0.0001 ***	0.019 *	<0.0001 ***	0.003 **
L2	97.1 ± 9.3	116.1 ± 22.7	77.5 ± 9.8	<0.0001 ***	<0.0001 ***	<0.0001 ***	<0.0001 ***
L3	88.8 ± 22.5	100.7 ± 26.2	42.2 ± 11.4	<0.0001 ***	0.032 *	<0.0001 ***	<0.0001 ***
One-way ANOVA	<0.0001 ***	<0.0001 ***	<0.0001 ***				
L1 vs. L2 (*p*)	<0.0001 ***	0.001 **	<0.0001 ***				
L1 vs. L3 (*p*)	<0.0001 ***	<0.0001 ***	<0.0001 ***				
L2 vs. L3 (*p*)	0.037 *	0.006 **	<0.0001 ***				
IL-10 (pg/mL)							
L1	10.9 ± 2.1	12.1 ± 2.2	14.1 ± 3.1	<0.0001 ***	0.010 *	<0.0001 ***	0.002 **
L2	9.4 ± 1.5	10.5 ± 1.8	7.2 ± 1.4	<0.0001 ***	0.006 **	<0.0001 ***	<0.0001 ***
L3	8.7 ± 1.8	9.6 ± 1.8	5.1 ± 1.5	<0.0001 ***	0.021 *	<0.0001 ***	<0.0001 ***
One-way ANOVA	<0.0001 ***	<0.0001 ***	<0.0001 ***				
L1 vs. L2 (*p*)	<0.0001 ***	<0.0001 ***	<0.0001 ***				
L1 vs. L3 (*p*)	<0.0001 ***	<0.0001 ***	<0.0001 ***				
L2 vs. L3 (*p*)	0.047 *	0.045 *	<0.0001 ***				

IL: interleukin; TNF-α: tumor necrosis factor alpha; L1: lot 1; L2: lot 2 with propofol; L3: lot 3 with propofol combined with fentanyl; T_0_: at the beginning of the procedure and administration of anesthetic drugs; T_1_: at 15 min after the beginning of the endoscopic procedure; T_2_: at 2 h after the end of the colonoscopy; * *p*-value < 0.05; ** *p*-value < 0.01; *** *p*-value < 0.0001.

## Data Availability

The data used to support the findings of this study are available from the corresponding author upon reasonable request.
